# Improving Left Atrial Appendage Occlusion Device Size Determination by Three-Dimensional Printing-Based Preprocedural Simulation

**DOI:** 10.3389/fcvm.2022.830062

**Published:** 2022-02-16

**Authors:** William D. Kim, Iksung Cho, Young Doo Kim, Min Jae Cha, Sang-Wook Kim, Young Choi, Seung Yong Shin

**Affiliations:** ^1^College of Medicine, Chung-Ang University, Seoul, South Korea; ^2^Division of Cardiology, Severance Hospital, Yonsei University College of Medicine, Seoul, South Korea; ^3^Department of Mechanical Engineering, Graduate School, Chung-Ang University, Seoul, South Korea; ^4^Department of Radiology, Chung-Ang University Hospital, Seoul, South Korea; ^5^Division of Cardiology, Chung-Ang University Hospital, Seoul, South Korea; ^6^Heart Research Institute, Chung-Ang University Hospital, Seoul, South Korea

**Keywords:** atrial fibrillation, left atrial appendage occlusion, three-dimensional printing, simulation, cardiac computed tomography

## Abstract

**Background:**

The two-dimensional (2D)-based left atrial appendage (LAA) occluder (LAAO) size determination by using transesophageal echocardiography (TEE) is limited by the structural complexity and wide anatomical variation of the LAA.

**Objective:**

This study aimed to assess the accuracy of the LAAO size determination by implantation simulation by using a three-dimensional (3D)-printed model compared with the conventional method based on TEE.

**Methods:**

We retrospectively reviewed patients with anatomically and physiologically properly implanted the Amplatzer Cardiac Plug and Amulet LAAO devices between January 2014 and December 2018 by using the final size of the implanted devices as a standard for size prediction accuracy. The use of 3D-printed model simulations in device sizing was compared with the conventional TEE-based method.

**Results:**

A total of 28 cases with the percutaneous LAA occlusion were reviewed. There was a minimal difference [−0.11 mm; 95% CI (−0.93, 0.72 mm); *P* = 0.359] between CT-based reconstructed 3D images and 3D-printed left atrium (LA) models. Device size prediction based on TEE measurements showed poor agreement (32.1%), with a mean difference of 2.3 ± 3.2 mm [95% CI (−4.4, 9.0)]. The LAAO sizing by implantation simulation with 3D-printed models showed excellent correlation with the actually implanted LAAO size (*r* = 0.927; bias = 0.7 ± 2.5). The agreement between the 3D-printed and the implanted size was 67.9%, with a mean difference of 0.6 mm [95% CI (−1.9, 3.2)].

**Conclusion:**

The use of 3D-printed LA models in the LAAO size determination showed improvement in comparison with conventional 2D TEE method.

## Introduction

Atrial fibrillation (AF) is associated with about five fold increased risk of ischemic stroke and is also responsible for 15% for all the strokes; moreover, it may lead to severe disability or even death 120 (1–3). The left atrial appendage (LAA), an accessory pouch-like structure attached to the main body of the left atrium (LA), is the main source (90%) of thrombus formation in patients with AF (2, 4). The standard treatment recommended for preventing stroke in these patients is oral anticoagulants (OACs), based on their CHADS_2_ or CHA_2_DS_2_-VASc score (3). However, there are patients with contraindications to anticoagulant therapy or recurring stroke during OAC treatment. For these cases, the epicardial or endocardial LAA closure has emerged as a valid alternative (4).

The percutaneous LAA occluders (LAAOs) are minimally invasive endocardial devices that have shown non-inferior effectiveness in stroke prevention with a marked reduction in bleeding risk (5–7). Comprehensive preimplantation assessment of the LAA anatomy is critical for procedural success and treatment outcomes in the percutaneous LAAO procedure (7–9). Traditionally, transesophageal echocardiography (TEE)-based two-dimensional (2D) imaging parameters including the diameter of landing zone, depth, orientation of the main anchoring lobe, and the number and origin of additional lobes have been used for the determination of the LAAO device type and size (7, 9). However, given the complexity and wide variation of the LAA structure, the current 2D TEE-based preimplantation assessment strategy shows limitations in device sizing accuracy and overall understanding of the three-dimensional (3D) LAA structure (4, 10).

Three-dimensional printing (3DP) has emerged as a new method for visualizing image models and its application in the medical field is also growing (11–13). In minimally invasive cardiac procedures, CT-based 3D-printed cardiac models have shown optimal implantation results for transcatheter aortic valve implantation in patients with aortic stenosis (AS) (14, 15). Yet, there is still lack of data in the application of 3DP to the LAAO, whereas it has the potential benefit of allowing precise evaluation of the LAA structure of an individual, leading to optimal device sizing with minimal implantation attempts. Therefore, we set out to assess the accuracy of the LAAO size determination method by implantation simulation by using a 3D-printed model compared to the conventional 2D TEE-based method.

## Materials and Methods

### Patient Enrollment

We retrospectively reviewed 57 cases with the percutaneous LAA occlusion by using the Amplatzer Cardiac Plug (ACP) and Amulet (Abbott Vascular Incorporation, Santa Clara, California, USA) from 2014 to 2018 at Chung-Ang University Hospital, South Korea. Exclusion criteria included the following: (1) cases without cardiac CT or CT images acquired by prospective gating leading to difficulty in assessment of the LA in end-systole images (23 cases) and (2) significant peridevice leakage defined by a color Doppler flow > 3 mm in width or inappropriate position of the device at the 6-month follow-up TEE (6 cases). We finally included 28 cases with the anatomically and functionally successful LAAO device implantation and used the successfully implanted device size as a gold standard for comparing the size prediction ability between the 2D TEE image and 3D-printed simulation method. The Institutional Review Board of Chung-Ang University Hospital approved this study and waived the informed consent requirements due to the retrospective nature of this analysis (IRB No. 1808-007-16198).

### Transesophageal Echocardiography Image Acquisition and Device Size Selection

Preprocedural TEE was performed in all the patients on the same day of the LAAO procedure to assess the LAA size, shape, and presence of thrombus. Patients were hydrated with intravenous 500 ml half-saline to achieve an LAA pressure suitable for the LAAO procedure. TEE was performed and analyzed by using the Philips iE33 (Philips Healthcare, Cleveland, Ohio, USA) device. The LAA was imaged at 0, 45, 90, and 135° on 2D TEE. The LAA ostium diameter referred for device size selection was defined as the line from the Coumadin ridge to the left circumflex coronary artery (16). The landing zone diameter was measured 1 cm distal and at a parallel line to the ostium. The device size selection for both the ACP and Amulet was primarily based on the landing zone diameter (7). The longest diameter of the landing zone that was usually measured at 90-135° were used for the size selection (17). The sizing chart provided by the device company was used for the device size selection (16).

### Preprocedural CT Image Acquisition

Preprocedural CT was performed by using the Philips Brilliance 64 CT Scanner (Philips Healthcare, Cleveland, Ohio, USA): slice collimation = 64 × 0.625, tube voltage = 120 kV, gantry rotation time = 270 ms, and retrospective ECG gating with ECG-based tube current modulation. Prior to coronary CT angiography, patients with a heart rate > 65 bpm were administered oral ß-receptor blockers (atenolol 50 mg; Tenormin^®^, AstraZeneca, Stockholm, Sweden) and every patient received 0.8 mg of nitroglycerin sublingually. Using the bolus tracking technique (Bolus Pro Ultra; Philips Healthcare, Cleveland, Ohio, USA), the contrast-enhanced scanning was initiated after 10 s of triggering, with a trigger threshold of 110 Hounsfield units in the ascending aorta. Approximately, 50–70 ml (Iomeron 400, 400 mg iodine/ml; Bracco Imaging SpA, Milan, Italy) of contrast agent was injected through an antecubital vein (injection rate = 4.5–5 ml/s) followed by a 50-ml 1:1 mixed contrast saline chaser (4 ml/s) by using a dual-head power injector (Stellant; Medrad Incorporation, Pittsburgh, Pennsylvania, USA). Images were reconstructed at end-diastolic (73–83% of the R-R interval) and end-systolic (30–40% of the R-R interval) phases, with a 20-cm field of view, 512 × 512-pixel matrix, 0.9 mm slice thickness, and 0.45 mm image increments with hybrid iterative reconstruction (iDose4; Philips Healthcare, Cleveland, Ohio, USA) by using medium soft-tissue convolution kernel. To assess the presence or absence of the LAAO thrombus, we also performed delayed scanning. All the CT scans were acquired with application of automatic arrhythmia detection system, which automatically detects irregular ECG events during a cardiac CT scan such as Premature ventricular contraction (PVCs) and Premature atrial contraction (PACs).

### Three-Dimensional Printing and Validation of the LAA Printing Model

**Figure 1:**
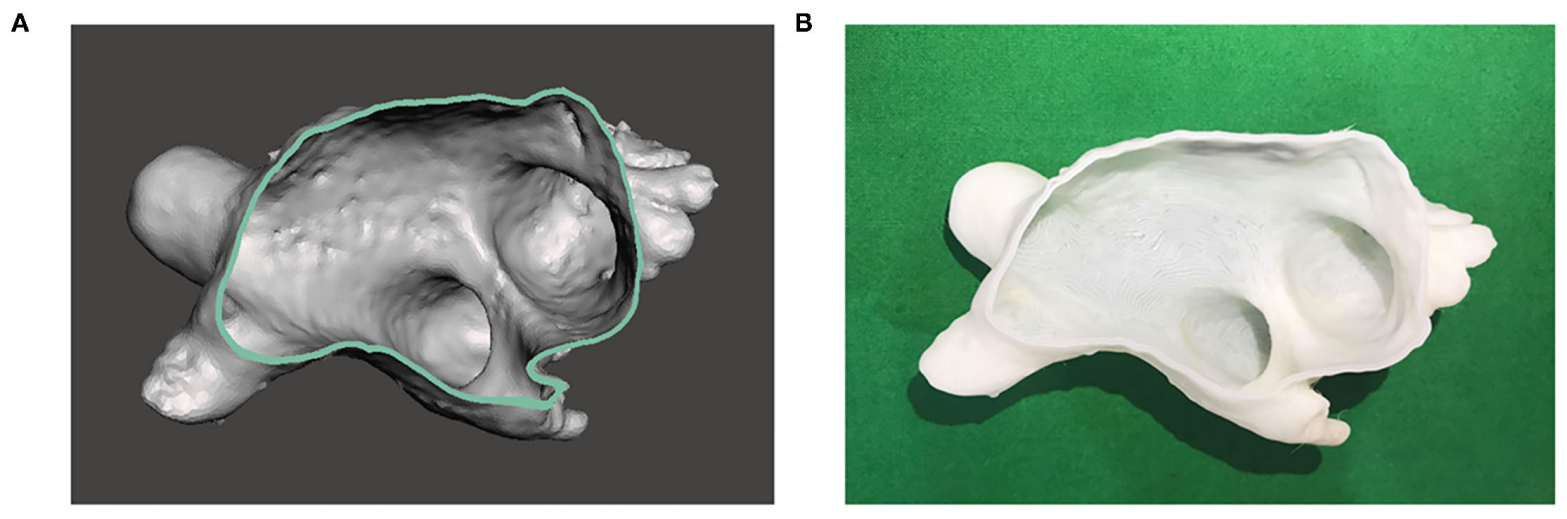
Three-dimensional (3D)-printed model of the left atrium (LA). **(A)** 3D image of the LA is reconstructed based on cardiac CT images. **(B)** 3D model of the LA is printed based on the image.

Computed tomography images were imported and confirmed on the 3D modeling software platform in the Digital Imaging and Communications in Medicine (DICOM) file format (AVIEW Modeler 1.0; Coreline Soft Incorporation, Seoul, Republic of Korea) (18–20). The blood volume of the target LA was segmented to create a sculpture and holes were filled to make a 3D model in Standardized Tessellation Language (STL) file format. The model was exported to another software platform (Meshmixer; Autodesk Incorporation, San Rafael, California, USA) to be smoothed. The final LA model was created by offsetting boundary with 2-mm thickness outward to represent the LA wall. Then, the model was printed by using the fused deposition modeling (FDM) machine (Dimension SST 768; Stratasys Incorporation, Rehovot, Israel) with P400 ABS material (Figure 1).

**Figure 2:**
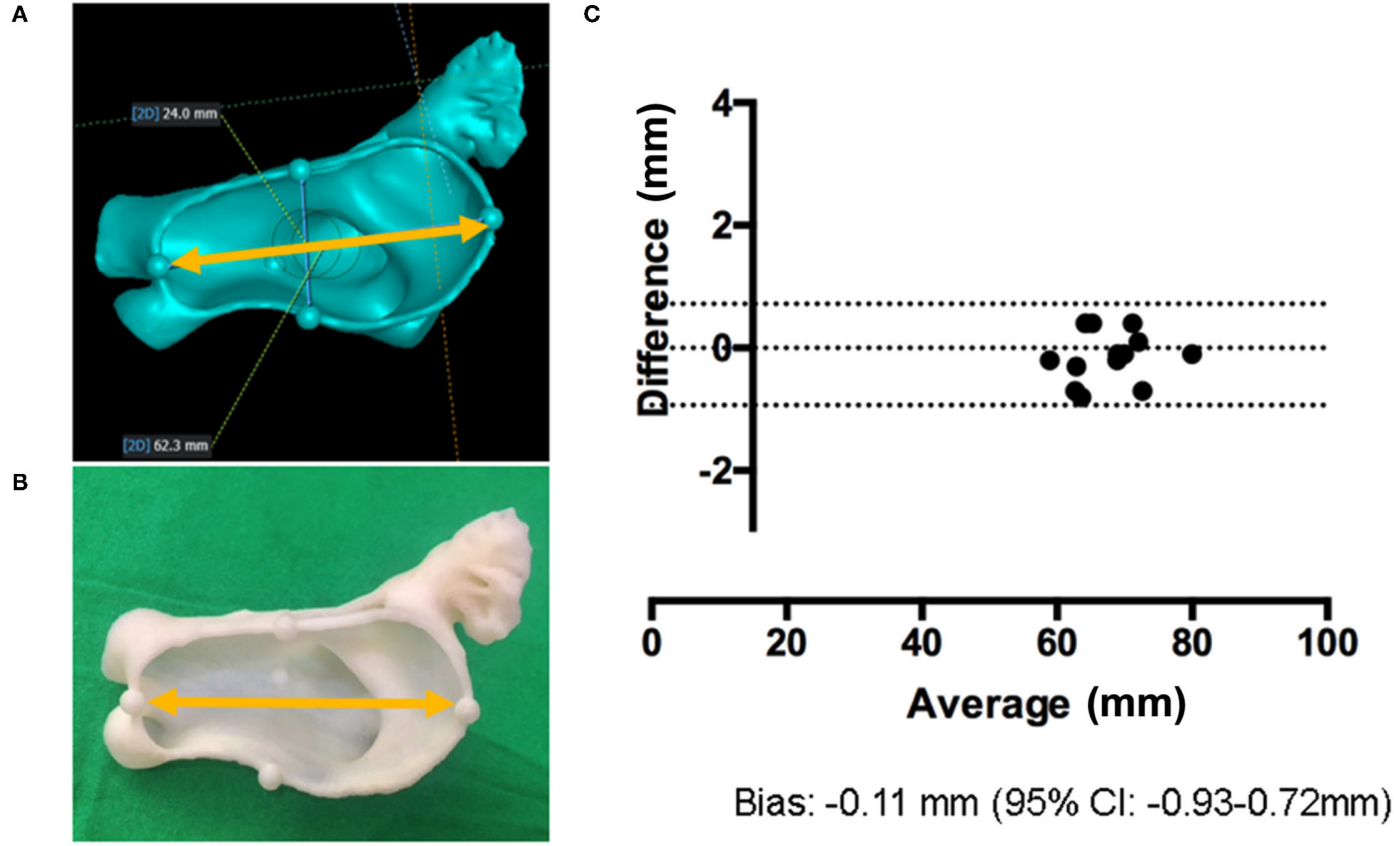
Validation of the 3D-printed LA model. **(A)** Artificial markings are added to 3D image and the distance between them is measured (yellow arrow). **(B)** The horizontal diameter in 3D-printed LA models is measured (yellow arrow). **(C)** The Bland–Altman plot comparing the horizontal diameter of 3D images and 3D-printed models.

The process mentioned above may cause an accuracy issue of the shape, due to the printing resolution of the FDM machine, changes in temperature, the characteristics of the used materials, or other factors (21). Therefore, we evaluated the accuracy of the printed 3D model size compared with CT images by measuring the distance between intentionally inserted artifacts to the LA model during image processing. As depicted in Figures 2A,B, the artifacts were created in the 3D LAA models by using commercial software (MAGICS RP; Materialize NV, Leuven, Belgium). The LA models, including the LAA, were printed and the horizontal diameter between the artifacts was measured in both the 3D image and the printed model. We evaluated the agreement between the distances measured on 3D model and on the printed model.

### Prediction of the LAAO Size by 3D-Printed Simulation or TEE

The LAAO size was determined by using conventional 2D TEE measurements by two experienced cardiologists who were blinded to the size of the implanted device. We defined the diameter of the landing zone at 10 mm distal to the ostium at an angle perpendicular to the ostial line, which was defined as the line from the Coumadin ridge to the left circumflex coronary artery. We measured the landing zone diameter at 0, 45, 90, and 135° images of 2D TEE. A device sizing chart for each device (ACP and Amulet) was utilized to select the proper size (16).

**Figure 3:**
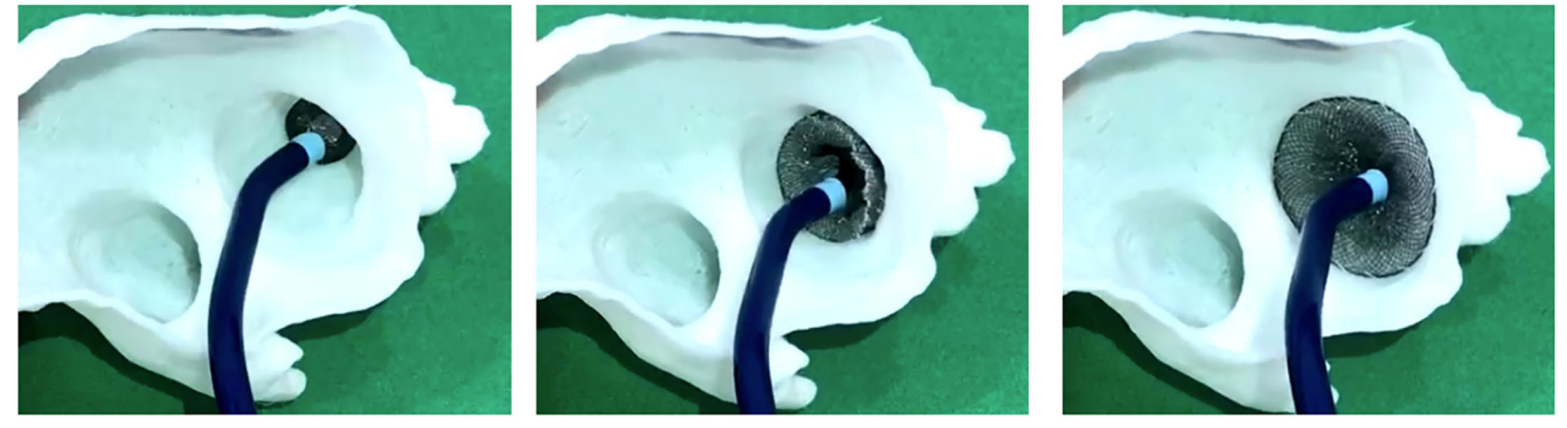
Left atrial appendage occluder implantation simulation by using a 3D-printed model.

The LAAO size was also independently determined by device implantation simulation by using the 3D-printed LAA model (Figure 3). The device implantation simulation was performed as follows. First, we determined the simulation device size by measuring the landing zone measurement by using 2D CT imaging. We defined the ostium and landing zone by using the same 2D TEE method at the end-systolic phase. However, we did not use the sizing chart for the device size determination, since it was mainly derived from echocardiographic images, which underestimates the inner diameter of cardiac structures. Furthermore, the interventionist, who was blinded to the size of implanted device or those predicted by TEE, implanted the LAAO device into the printed LAA at the most proper location and performed a “tug test” for confirmation of stability. We determined the proper LAAO size according to the following criteria: (1) no significant gap (more than 3 mm) between the implanted LAAO device and the printed LAA; (2) the concave-shaped LAAO device disk; and (3) the LAAO device should not be retracted or embolized during the “tug test.” To select the ideal LAAO size, we increased or decreased the size of the implantation simulation device from the initial tested size until the criteria were best fulfilled.

### Statistical Analysis

The agreement between the size of the printed LAA 3D model and that of CT images by using intentionally inserted artifacts to the LA model was evaluated with the Bland–Altman analysis. Furthermore, the correlation and agreement between 2D TEE-determined and the actual implanted device size were evaluated with the Pearson's correlation analysis and the Bland–Altman analysis, respectively. We also assessed the correlation and agreement between the device size determined by simulations by using 3D-printed model and the device size successfully implanted with the Pearson's correlation coefficient and the Bland–Altman analysis, respectively. All the tests were two-sided; *P*-values < 0.05 were regarded as statistically significant. Analyses were performed by using MedCalc version 14.12.0 (MedCalc Software Incorporation, Mariakerke, Belgium, UK).

## Results

### Baseline Characteristics

Baseline clinical characteristics of patients included in this study are shown in Table 1. Overall, the study population consisted of 28 individuals with the successful LAAO implantation; mean age was 73 ± 11 years and 16 (57.1%) were male. Previous stroke during anticoagulation and major/minor bleeding were present in 13 (46.4%) and nine patients (32.1%), respectively. The mean CHA_2_DS_2_-VASc and HAS-BLED scores were 4.3 ± 2.2 and 3.5 ± 1.7, respectively. Amulet and the ACPs were implanted in 16 (57.1%) and 12 (42.9%) patients, respectively.

**Table 1:** Baseline clinical characteristics.

**Characteristics**	**Value**
Age	73 ± 11
Sex, male	16 (57.1%)
Body mass index	24.0 ± 3.2
Hypertension	26 (92.3%)
Diabetes mellitus	7 (25.0%)
Heart failure	6 (21.4%)
Stroke	13 (46.4%)
Coronary artery disease or peripheral artery disease	6 (21.4%)
Major/minor bleeding	9 (32.1%)
CHA_2_DS_2_VASc score	4.3 ± 2.2
HAS-BLED score	3.5 ± 1.7
Device, Amulet	16 (57.1%)
Device, Amplatzer cardiac plug	12 (42.9%)

### Validation of the 3D-Printed LAA Model Size

As shown in Table 2, the difference in the diameter between the artifacts was non-significant (*P* = 0.359). The Bland–Altman analysis confirmed this minimal difference [−0.11 mm; 95% CI (−0.93, 0.72 mm)] between the 3D images and printed models (Figure 2C).

**Table 2:** Differences in size of left atrium by three-dimensional (3D) images and 3D-printed model.

	**3D imaging**	**3D printed model**	**Mean difference**	***P*-value**
Diameter size, mm	67.46 ± 5.48	67.57± 5.45	−0.11 ± 0.42	0.359

*P-value for paired t-test*.

### Comparison of the LAAO Sizing Accuracy Between 2D TEE Measurements and the Implanted Size

**Figure 4:**
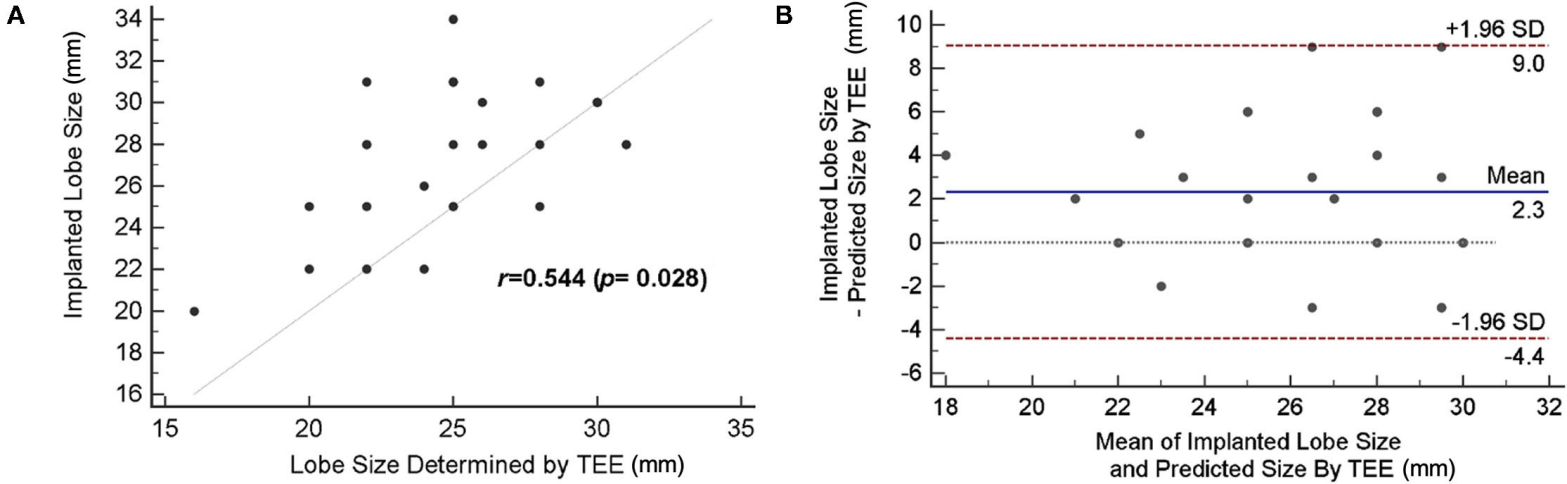
Accuracy of device size prediction based on transesophageal echocardiography (TEE). **(A)** Correlation of TEE-based predicted size and actually implanted lobe size. **(B)** The Bland–Altman plot comparing the predicted size by TEE and actual device size.

Figure 4A demonstrates the correlation between the lobe size of device determined by 2D TEE and actual implanted size. There was poor agreement (*r* = 0.544) when using 2D TEE as the imaging modality for device size selection. The exact agreement between the size suggested by TEE and the implanted size was 32.1%, with 16 cases (57.1%) of underestimation and three cases (10.7%) of overestimation when device sizing was based on TEE (Table 3). The mean difference between implanted and predicted lobe size by using TEE was 2.3 mm [95% CI (−4.4, 9.0)] (Figure 4B), showing an underestimation of the LAAO device size.

**Table 3:** Predicted and implanted left atrial appendage occluder device sizes.

**Case number**	**Implanted device lobe size, mm**	**Predicted size by TEE, mm**	**Difference**	**Predicted size by 3DP Simulation, mm**	**Difference**
1	30	30	0	28	−2
2	22	22	0	22	0
3	28	26	−2	28	0
4	30	30	0	30	0
5	26	24	−2	28	2
6	20	16	−4	20	0
7	28	22	−6	28	0
8	30	26	−4	28	−2
9	22	24	2	20	−2
10	30	30	0	30	0
11	28	22	−6	26	−2
12	30	30	0	30	0
13	22	20	−2	22	0
14	25	20	−5	25	0
15	25	25	0	25	0
16	31	25	−6	31	0
17	31	28	−3	31	0
18	31	28	−3	28	−3
19	31	22	−9	28	−3
20	28	28	0	28	0
21	25	28	3	22	−3
22	25	22	−3	25	0
23	34	25	−9	34	0
24	28	31	3	28	0
25	28	25	−3	28	0
26	25	25	0	25	0
27	28	28	0	28	0
28	31	25	−6	28	−3
Percentage of cases with accurate size prediction		32.1%		67.9%	

*TEE, transesophageal echocardiography; 3DP, three-dimensional printing*.

### Comparison of the LAAO Sizing Accuracy Between the Simulation With 3D Printing and the Implanted Size

**Figure 5:**
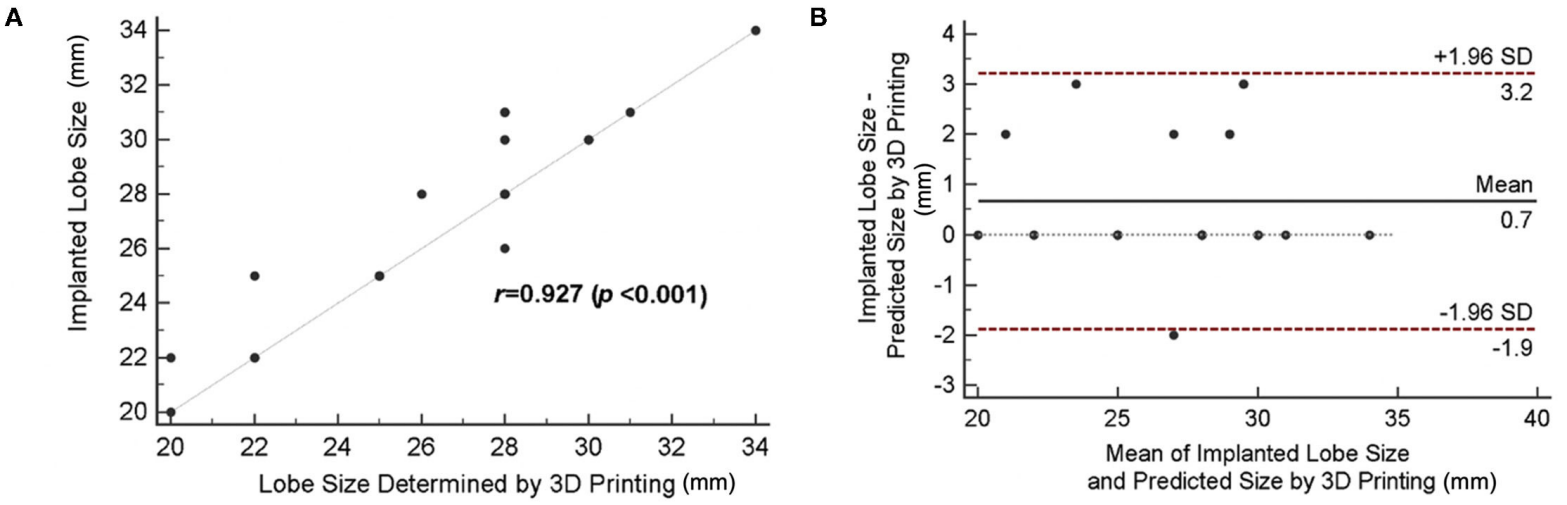
Accuracy of device size prediction based on 3D printing simulation. **(A)** Correlation between 3D-printed simulation-based predicted size and actually implanted lobe size. **(B)** The Bland–Altman plot comparing the predicted size by 3D printing simulation and the actual device size.

Compared with the 2D TEE method, an improved correlation (*r* = 0.927; bias = 0.7 ± 2.5) was shown when device size prediction was made with 3D-printed models (Figure 5A). The mean difference between the implanted and predicted lobe size by using 3D-printed models was 0.7 mm [95% CI (−1.9, 3.2)] (Figure 5B). As demonstrated in Table 3, the agreement between the size suggested by the 3D-printing simulation and the implanted size was 67.9%, with eight cases (28.5%) of underestimation and only 1 case (3.6%) of device size overestimation. When comparing the sizing method based on 3D-printing simulation with the TEE method, the mean difference was 1.7 ± 3.3 mm (*P* = 0.012).

## Discussion

In this study, we found a superior accuracy of device size simulation by using 3D-printed models of the LAA compared with the conventional 2D TEE method. Predicted device size based on 3D-printing simulation showed high accuracy, with 67.9% of patients matching the actually implanted LAAO devices. 2D TEE-based size determination showed poor agreement and general undersizing, with a match seen in only 32.1% of patients.

For patients who are contraindicated for OAC treatment or cannot tolerate long-term OAC use, the LAAO is an alternative treatment option to prevent strokes and is becoming more widely used (22, 23). For the successful LAAO implantation, proper size determination of the implanted device is one of the most critical preprocedural steps. However, the current traditional size prediction system by using 2D TEE showed substantial discrepancy and limitations. For instance, in a previous study by Clemente et al., 2D TEE significantly underestimated the landing zone diameter; thus, the agreement between the suggested and used size of the LAAO device was as low as 5.9% (4). The agreement was improved by using 2D CT imaging, but remained at only 32.4% compared with the implanted devices. In another study performed in 37 patients with AF, the size predicted by 2D TEE measurements was the same as that of the actually implanted device only in 51.4% of patients (24). In this study, prediction by TEE resulted in 7 cases with 2-size error (25%) and 10 cases with 1-size error (35.7%), while all the cases with mismatch by 3D printing-based prediction (32.1%) had only 1-size error.

The main reasons why the conventional 2D-based imaging methods used for the LAAO device sizing show poor accuracy are the following: (i) the complex overall anatomical structure of the LAA and (ii) the diverse shape and size of the LAA ostium (25, 26). Thus, the ostium, landing zone, and the LAA depth measurements based on conventional 2D methods may lead to mismatch in device size, possibly provoking adverse procedural events. 3D imaging by using TEE or cardiac CT has been emerging as an important technique, significantly improving visualization of the complex anatomy of the cardiovascular system (4, 27, 28). However, it shows imperfect accuracy when predicting the interaction between the LAAO device and its surroundings during and after implantation (10). 3D printing based on 3D imaging has advantages in this aspect, enabling realistic procedural simulation and printed models of the LAA and, thus, optimizing the choice of device and procedural processes. There are substantial and variable angulations between the landing zone and ostium; thus, the center of disk may be placed eccentrically, which may result in incomplete closure. These conformational changes caused by complex interactions between lobes and ranges of landing zones can only be assessed by 3D printing-based realistic preprocedural simulations. With this approach, improved procedural safety may be achieved by minimizing the number of implantation attempts.

In this context, only a few studies have shown attempts to use 3D printing to assess the complex LAA structure. Otton et al. (29) first introduced the use of 3D printing in the LAAO procedures and several studies (30–33) have since validated its use. However, in those studies, uncertainty in the optimal size of the actually implanted devices remained. In this study, we used cases with the successfully implanted Amplatzer or Amulet confirmed by follow-up TEE imaging at 6 months after the procedure, thus being able to establish a gold standard for applying 3D printing in the LAAO.

The choice of material used in 3D printing may be an important factor, as the heart is a dynamic organ, consistently cycling through systole and diastole. Elasticity, density, asperity, and other characteristics of the material closely resembling the tissue of the heart would be ideal when performing simulation with printed models (15). However, distortion of the printed product may occur as temperature changes that cause contraction of the material. In this study, we used a hard material, thus minimizing this error and allowing us to obtain 3D-printed products with high proximity to the imaging-based models. However, the limitation of using a hard material for simulation would be the imperfect reflection of the structural changes in the LAA or material interaction between the occluder device and the LAA tissue. In further studies, it may be necessary to apply models fabricated from flexible materials in the LAAO simulations. However, considering the cost and feasibility of 3D printing with advanced materials in practicing centers, the practicality of this method may not yet be as cost-effective.

Another issue would be the selection of patients to whom 3D printing simulation would be most beneficial. Although advances in 3D printing technology and equipment have made it easier to print models of individualized hearts, the 3D printing and simulation process are associated with an additional cost and greater time consumption. Therefore, the application of 3D printing in the LAAO procedure should be reserved to selected cases with expected sizing or procedural difficulties. Possible cases of sizing difficulty would be those with the LAA thrombi, eccentric ostium, extremely the large or small LAA, the LAA with multilobes or proximal bifurcation, and those with other structural complexities. Also, the location of septal puncture is an important step in the procedure and 3D-printed models with the right atrium included may enable preprocedural planning of it. Further studies should be performed to better define cases with size discrepancy between the current 2D imaging and implanted device as well as their anatomical characteristics.

There are a few limitations to this study. First, this study evaluated cases with the successfully implanted LAAO devices, leaving the possibility of cases being excluded due to procedural failure or complication due to TEE sizing inaccuracy. This is an explorative study of feasibility of 3D printing-based prediction in the LAAO sizing, so we are now starting a prospective study to prove this concept in a more representative patient population. Furthermore, this study is a retrospective analysis and may have been influenced by unobserved confounders, selection or referral biases, or both. Thus, the clinical feasibility and usefulness of 3D printing-based simulation method, such as improved success rate, decreased procedure time or dose of contrast and radiation, must be verified in future prospective studies. Third, this study only included cases that used the ACP or Amulet. Therefore, the feasibility and accuracy of the LAAO device sizing by 3D printing-based simulation could not be applied to other devices, such as Watchman. The exclusion of Watchman devices in this study was based on the different LAA evaluation and sizing methods. Fourth, there was a lack of comparison with CT-based sizing and TEE or 3D printing-based sizing methods. Cardiac CT images allow accurate evaluation of the LAA anatomy (34) and as 3D printing is based on reconstructed CT images, it would be an important matter to include CT-based sizing methods for comparison in future studies. Last, the relatively small sample size should also be taken into consideration in this study. We plan to continue our research and conduct multicenter studies to overcome this limitation.

In conclusion, the LAAO size determination by using simulation with 3D-printed models of the LA showed improved accuracy compared with conventional methods based on 2D TEE. Future prospective studies evaluating the clinical utility of the 3D printing-based size determination method should be performed.

## Data Availability Statement

The original contributions presented in the study are included in the article/supplementary material and further inquiries can be directed to the corresponding author/s.

## Ethics Statement

The studies involving human participants were reviewed and approved by Institutional Review Board of Chung-Ang University Hospital. Written informed consent for participation was not required for this study in accordance with the national legislation and the institutional requirements.

## Author Contributions

IC, WK, and SS participated in the design of the study, acquisition of data, statistical analysis, interpretation of data, and drafting of the manuscript. YK and YC carried out the 3D printing of cardiac models. MC participated in the analysis and interpretation of cardiac imaging data. S-WK and SS carried out the LAAO intervention procedures. All authors participated in the final revision of the manuscript.

## Funding

This study was supported by a Severance Hospital Research fund for Clinical excellence (SHRC) (C-2020-0041) and a faculty research grant of Yonsei University College of Medicine (6-2020-0156).

## Conflict of Interest

The authors declare that the research was conducted in the absence of any commercial or financial relationships that could be construed as a potential conflict of interest.

## Publisher's Note

All claims expressed in this article are solely those of the authors and do not necessarily represent those of their affiliated organizations, or those of the publisher, the editors and the reviewers. Any product that may be evaluated in this article, or claim that may be made by its manufacturer, is not guaranteed or endorsed by the publisher.
